# Differences in Physical Performance and Body Composition Between National and Non-National Youth Female Handball Players

**DOI:** 10.3390/sports14030089

**Published:** 2026-02-27

**Authors:** Bálint István Ruppert, Richárd Bauer, Bálint Kilvinger, Árpád Petrov, István Barthalos, László Suszter, Csaba Ökrös, Ottó Vincze, Antonio Ferraz, Zoltán Alföldi, Ferenc Ihász

**Affiliations:** 1 Doctoral School of Regional and Economic Sciences, Széchenyi István University, H-9026 Győr, Hungary; bauerrc79@gmail.com (R.B.); kilvingerbalint@gmail.com (B.K.);; 2 Department of Health Promotion and Exercise Science, Széchenyi István University, H-9026 Győr, Hungaryalfoldi.zoltan@sze.hu (Z.A.); ihasz.ferenc@sze.hu (F.I.); 3 Sport and Health Sciences Research Group, Eszterházy Károly Catholic University, H-3300 Eger, Hungary; 4 Sports Games Department, Hungarian University of Sport Science, H-1123 Budapest, Hungary; 5 Doctoral School of Health Sciences, University of Pécs, H-7624 Pécs, Hungary; 6 Center for Sports Research and Training, Sports Research, and Training Center, Jean Piaget University of Angola, Viana B.P. 681, Angola; antferraz@hotmail.com

**Keywords:** handball, conditioning, body composition, selection

## Abstract

Performance differences between female youth handball players selected for national teams and non-selected peers are often linked to strength, speed, and power. This study aimed to compare the conditioning capacities and body composition of national and non-national youth handball players. The sample included 36 female players (17.13 ± 1.75 years), 18 national and 18 position-matched non-national players. Anthropometry, sprint and change in direction ability, vertical jump, upper- and lower-body strength, aerobic capacity, and body composition were assessed using standard tests and bioimpedance analysis. For normally distributed variables, an independent-samples *t*-test was applied, while for variables that did not meet the normality assumptions, the Mann–Whitney U test was used. Cohen’s d was used to assess effect size. National team players showed significantly greater jump height (*p* < 0.001, d = 1.408), higher relative peak power (*p* < 0.001, d = 1.644), and faster 20 m sprint times (*p* = 0.004, d = −1.037). No significant differences were found in body composition or the other measured parameters, although a moderate Yo-Yo IRL1 effect size suggests a potential practical advantage in aerobic capacity for national team players. These results indicate that explosive power and linear speed are key discriminators for youth national-team selection.

## 1. Introduction

Handball is a high-intensity, contact sport that requires a high level of both aerobic and anaerobic performance [1]. Several studies have confirmed that, in addition to tactical and technical skills, a high level of conditional abilities and anthropometric characteristics are also performance-influencing factors in handball [2,3]. The physical fitness of players can greatly influence the success of the team [4]. Body height, body mass, and body composition play a decisive role in physical performance and are important factors in athlete selection [5]. In addition, data derived from motor tests (strength, speed, endurance) can be reliable predictors of performance; therefore, they hold particular significance in the evaluation of athletes [6]. In various sports–including handball–it is of key importance to identify, through objective performance indicators, those physical abilities that distinguish elite athletes from those who are less outstanding.

When examining the performance of handball players, particular attention is given to test protocols assessing physical abilities, which include jumping, sprinting, agility and endurance [7,8,9]. Several studies have demonstrated that the countermovement jump and squat jump tests possess high validity [9,10], and that Yo-Yo IR tests are suitable for determining intermittent endurance [7,11]. Tests assessing strength and explosiveness are essential among handball players [12]. In light of this, the use of these tests is scientifically well-founded and important for examining the physical demands of the sport.

In youth sports, the identification of talented players represents a complex, multifactorial process in which objective metrics constitute a critical component, complementing technical, tactical and psychological determinants of performance [13,14]. In handball-specific physical attributes, namely maximal strength, speed and power are recognized as key indicators in the athlete selection process, given their direct impact on sport-specific actions such as jumping, running, sprinting and physical-contact situations [15,16]. Nevertheless, the contribution of specific physical abilities to performance may differ according to age, competitive level and sex with limited empirical evidence currently available for female youth athletes [17,18]. A comprehensive understanding of the physical performance characteristics that differentiate youth national team handball players from their non-selected peers may facilitate the development of more objective, evidence-based selection criteria and simultaneously support the long-term athletic development of talented players [13].

One study investigated which tests reveal differences between handball players of various performance levels [19]. The greatest differences were recorded in explosive strength (i.e., squat jump and countermovement jump), in favor of the Olympic team. When comparing youth national team and non-national team handball players, members of the national team outperformed their peers in several key tests (standing long jump, 30 m sprint, maximal oxygen uptake) [20]. In young female handball players, significant performance differences were found between elite and non-elite groups, and significant differences were also observed in anthropometric and body composition variables [21]. Wagner et al. [22] demonstrated that elite male handball players exhibit greater jump performance, maximal muscle strength, and better throwing velocity. Although numerous studies have examined the conditional and anthropometric characteristics of handball players in adult samples, considerably fewer data are available regarding youth athletes, particularly concerning differences between national team and non-national team players.

The purpose of this study was to determine whether differences in physical performance and body composition exist between youth national team and non-national female handball players, and to identify which variables most strongly characterize attainment of youth national team level. Our results may support the development of talent identification systems and provide practical benefits for sports professionals in designing more targeted training programs.

## 2. Materials and Methods

### 2.1. Experimental Approach

Motor performance tests were conducted on 15 December 2025 at the Magvassy Mihály Sports Hall in Győr, Hungary. The test battery consisted of validated assessments supported by the scientific literature, and all procedures were executed according to established methodological guidelines [23,24,25,26]. On a separate occasion, on 16 December 2025, the athlete’s body composition was evaluated. Before data collection, athletes, their guardians, and the discipline-specific coaches received verbal instructions and subsequently provided written informed consent for participation in the study.

### 2.2. Participants

The study included 36 female youth handball players from the Győri Audi ETO KC Handball Academy (age: 17.13 ± 1.75 years), comprising 18 national team athletes (height: 171.35 ± 4.73 cm; body mass: 67.68 ± 6.22 kg) and 18 position-matched non-selected players (height: 172.06 ± 8.30 cm; body mass: 66.52 ± 7.72 kg). National team status was defined as having received at least two invitations to national youth team training camps and/or participation in age-group world championships. To ensure comparability, each national team player was matched with a non-selected player by playing position. On average, the athletes completed five handball training sessions, one competitive match, and two strength-training sessions per week, with no differences between the two groups in training frequency or match exposure.

**Figure 1:**
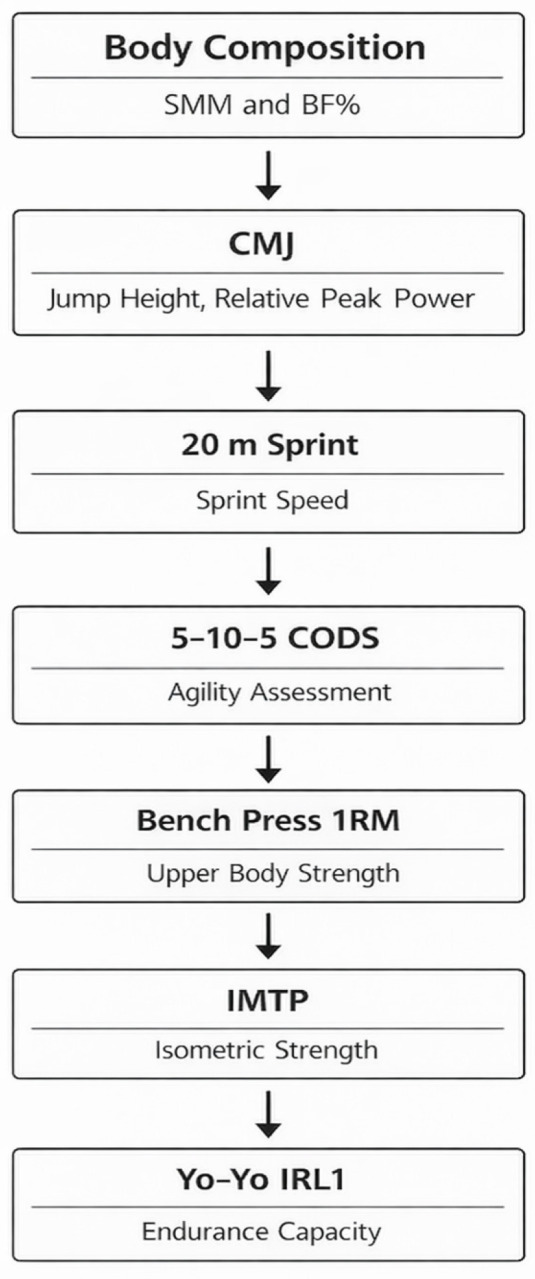
The schematic illustration of the measurement protocol. CMJ = countermovement jump, m = meter, CODS = change in direction speed, 1RM = one-repetition maximum, IMTP = isometric mid-thigh pull, IRL 1 = intermittent recovery level 1.

To minimize fatigue-related bias, athletes were instructed to refrain from strenuous training and competitive matches for 48 h before testing [27]. The sequence of testing was designed to reduce neuromuscular fatigue and maximize the validity of performance outcomes. According to the protocol defined by the Hungarian Handball Federation, testing commenced with explosive power and speed assessments–given their high sensitivity to fatigue–followed by high-force strength measures and concluded with endurance evaluation (Figure 1).

### 2.3. Measurement Protocol

Countermovement Jump (CMJ): The CMJ is a widely used method for assessing lower-limb force production, explosiveness, and stretch-shortening cycle efficiency [28]. Force plates are considered the “gold standard” for measuring jump performance, as kinetic variables derived from ground reaction force provide accurate estimates of jump height, force generation, and other parameters [23]. The assessment was performed using a Vald ForceDecks Dual Force Plate system (FD Lite model, Vald Performance, Newstead, Queensland, Australia; sampling frequency: 1000 Hz). Participants executed three maximal countermovement jumps with 30 s of rest between attempts. Athletes received verbal instructions prior to the test; verbal encouragement was not permitted during performance. For analysis, the highest recorded jump height (flight-time derived, cm) and relative peak power (W·kg^−1^) were included.

20 m Sprint Test: The first pair of Witty photocells (Microgate, Bolzano, Italy) was positioned at the start line, 1.5 m apart. The second pair was placed 20 m from the start. Athletes began from a standing start 40 cm behind the start line, without a flying start. Each athlete initiated the test voluntarily following a standardized instruction: to complete the sprint in the shortest possible time. Three maximal trials were performed with 3 min of passive rest between attempts. The fastest times (s) were used for analysis.

Change in Direction Speed (5-10-5 CODS): This test assessed players’ ability to change direction twice at 180°. Witty photocells were used for timing, with tripods opened to the first locking position and photocells placed above the midline. Cones were positioned 5 m to each side of the midpoint, and tape markings created parallel lines to ensure precise plant and cut execution, leaving a 40 cm gap at the center. Athletes assumed an athletic stance at the start line, positioned between both feet. After a self-initiated start, they sprinted left, planted the left foot at the cone while maintaining forward-facing orientation, returned through the start line, and repeated the movement to the right side. The test ended upon crossing the start line after the second change in direction. Players completed three trials to each side (left and right). For each direction, the fastest valid trial was selected. The final CODS performance value was calculated as the mean of the fastest left- and right-side trials (s). This approach was applied to minimize the influence of side dominance and to provide a representative measure of overall change in direction performance.

Bench Press Relative Maximal Strength: Upper-body maximal strength was determined using the one-repetition maximum (1RM) bench press test. The 1RM bench press test was conducted according to standardized research protocols, which prescribe a controlled movement pattern (lowering the barbell to the chest followed by a controlled concentric press), a progressive warm-up, standardized grip width, and maintenance of a stable body position [26,29]. Following a standardized warm-up, athletes performed sets of 1–3 repetitions with progressively increasing loads until reaching the maximal weight that could be lifted once with proper technique. The obtained 1RM (kg) was normalized to body mass to determine relative strength (1RM/body mass, kg·kg^−1^).

Lower-Limb Maximal Isometric Strength (Isometric Mid-Thigh Pull; IMTP): Lower-limb maximal force production was assessed using the IMTP test, a reliable measure of maximal isometric strength [24]. Athletes performed maximal isometric pulls with a bar positioned at mid-thigh height, defined as the midpoint between the iliac crest and the superior border of the patella. Bar height was individually adjusted using anatomical landmarks to ensure reproducibility. At the starting position, knee- and hip joint angles were standardized to approximately 125° and 145°, respectively, in accordance with established IMTP methodology (Comfort et al., 2019) [30]. The protocol followed international recommendations and Alex Natera’s standardized methodology, including standardized joint positioning, a progressive warm-up procedure, and maximal voluntary isometric contractions (Comfort et al., 2019) [30]. Before maximal attempts, participants completed three submaximal efforts (50%-75%-90% of estimated maximum) to minimize technical errors and reduce injury risk. Force production was measured using the Vald ForceDecks Dual Force Plate system (FD lite model, Vald Performance, Newstead, Queensland, Australia; sampling frequency: 1000 Hz). Absolute peak force (N) was recorded as the highest point of the force–time curve. For methodological consistency with the upper-body strength assessment, relative peak force was also calculated by normalizing absolute peak force to body mass (N·kg^−1^).

Yo-Yo Intermittent Recovery Test Level 1 (Yo-Yo IRL1): The Yo-Yo IRL1 is a widely applied test for assessing sport-specific aerobic capacity and recovery ability during repeated high-intensity efforts characteristic of intermittent sports [30]. Participants performed 2 × 20 m shuttle runs at progressively increasing speeds, with 10 s of active recovery between bouts. Running pace was controlled using audio signals (bleep test application). The test was terminated when an athlete failed to reach the finish line in time with the audio signal on two consecutive occasions. Total distance covered (m) was recorded.

Body Composition Assessment: Body composition was measured using an Accuniq BC720 multi-frequency bioelectrical impedance analyzer (Accuniq, Seoul, Republic of Korea), whose reliability and validity have been confirmed [25]. Measurements followed the manufacturer’s protocol. Athletes wore light sports clothing (shorts and a sports bra) and stood barefoot on the device while segmental impedance was measured. To ensure reliability, assessments were conducted in a fasted state and participants refrained from fluid intake during the hour preceding measurement. The variables recorded for analysis were total body mass (kg), skeletal muscle mass (SMM, kg) and body fat percentage (BF%). Height was measured using a validated portable stadiometer (Seca 213, Hamburg, Germany). Athletes stood barefoot with heels, scapulae, and head aligned to the stadiometer backboard, in a neutral head position with fully extended knees. Measurements were taken during a natural breath hold following inhalation, in accordance with anthropometric recommendations, with millimeter accuracy. This research was approved by the Istvan Szechenyi University Scientific Council (ETT) Committee on Scientific Ethics SZE/ETT-95/2025 (XII.5), Hungary.

### 2.4. Statistical Analysis

Statistical analyses were performed using JASP 0.16.0.0. (JASP Team, Amsterdam, Netherlands). Normal distribution of data was assessed using the Shapiro–Wilk test. Statistical significance was set a priori to *p* < 0.05. For variables showing normal distribution, an independent-samples *t*-test was used to determine group differences. When the assumption of normality was violated, the non-parametric Mann–Whitney U test was applied. Effect sizes (ES) were calculated using Cohen’s d, and were interpreted as very small (<0.20), small (0.20–0.59), moderate (0.60–1.19), large (1.20–1.99), and very large (>2.20) [31].

## 3. Results

The mean and standard deviation values of the test results and basic anthropometric characteristic of the examined groups are presented in Table 1.

**Figure 2:**
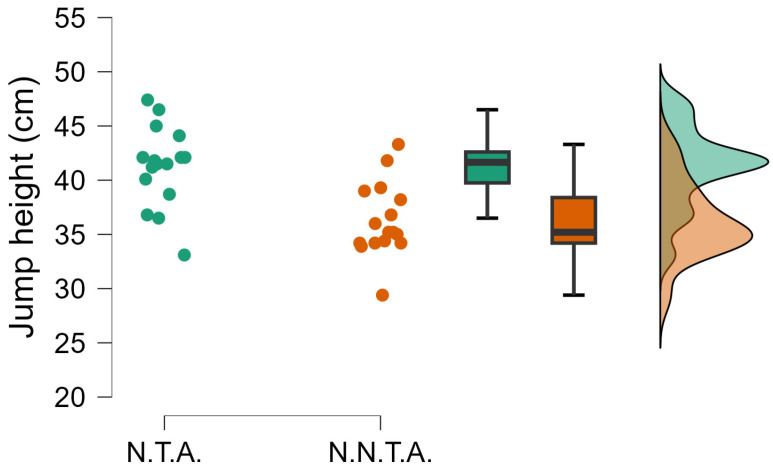
The significantly different jump height (t = 4.163; *p* < 0.001; Cohen’s d = 1.408). N.T.A. = selected athletes, N.N.T.A. = non-selected athletes.

**Figure 3:**
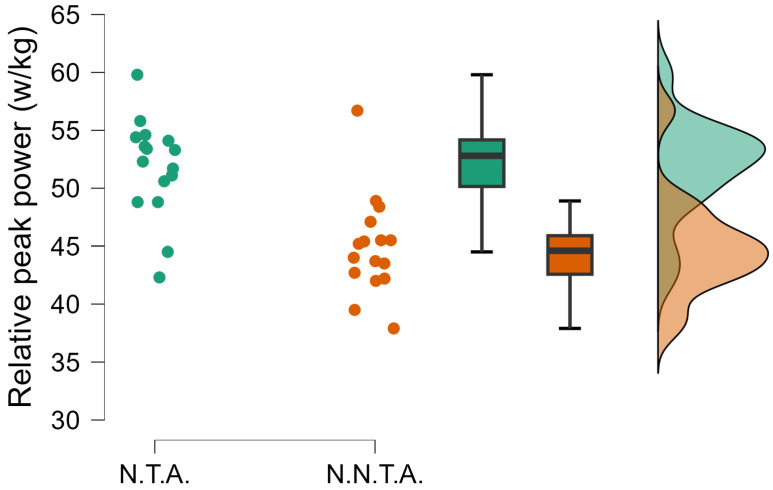
The significantly different relative peak power (t = 4.862; *p* < 0.001; d = 1.644). N.T.A. = selected athletes, N.N.T.A. = non-selected athletes.

**Figure 4:**
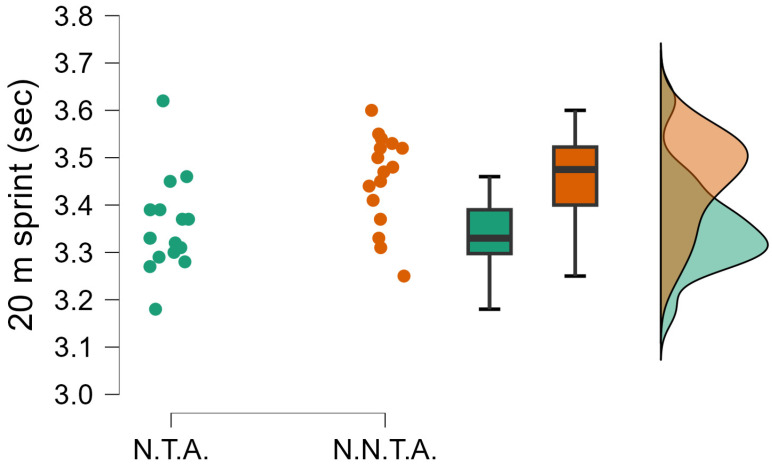
The significantly different 20 m sprint (t = −3.067; *p* = 0.004; d = −1.037). N.T.A. = selected athletes, N.N.T.A. = non-selected athletes.

The independent-samples *t*-test comparing the performance of selected and non-selected handball players showed significant differences in several variables. National team athletes achieved significantly greater jump height (t(df) = 4.163; *p* < 0.001; d = 1.408) (Figure 2), and their relative peak power was also substantially higher (t(df) = 4.862; *p* < 0.001; d = 1.644) (Figure 3). In the 20 m sprint time, the selected group proved to be faster (t(df) = −3.067; *p* = 0.004; d = −1.037) (Figure 4).

No significant differences were found between the groups for the other performance variables. In the 5-10-5 change in direction test (t(df) = −1.028; *p* = 0.312; d = −0.348), relative bench press (t(df) = 1.787; *p* = 0.083; d = 0.604), the Yo-Yo IRL1 endurance test (t(df) = 1.563; *p* = 0.013; d = 0.529), IMTP absolute (t(df) = 0.007; *p* = 0.994; d = 0.002), and relative values (t(df) = 0.525; *p* = 0.603; d = 0.178), no statistically detectable differences were observed. The anthropometric variables—body height (t(df) = −0.305; *p* = 0.762; d = −0.105), body mass (t(df) = 0.493; *p* = 0.625; d = 0.167), skeletal muscle mass (t(df) = 0.681; *p* = 0.501; d = 0.237), and body fat percentage (t(df) = −1.644; *p* = 0.110; d = −0.573)—also did not differ significantly between the two groups.

## 4. Discussion

This study aimed to identify differences in body composition and conditional performance between youth national team and non-national team female handball players. Our results indicate that the two groups differed significantly in several physical abilities that are considered crucial for handball performance. Specifically, we found differences in explosive strength assessed by the CMJ, as well as in linear speed (20 m sprint), with national team players outperforming their non-selected peers. Surprisingly, for most of the other motor tests, such as 5-10-5 CODS, IMTP, and relative maximal pushing strength (1RM bench press/body weight), no significant group differences were observed. Similarly, no meaningful differences emerged between groups in anthropometric characteristics (body height, body mass) or in body composition variables (BF%, SMM, kg).

One of the most prominent findings of our research was the significantly superior performance of national team players in the CMJ test. The greater jump height and higher relative peak power indicate that the selected players possess enhanced explosive strength which are key determinants of sport-specific performance in handball [32,33,34]. These findings align with previous studies reporting higher jump-performance among female handball players of varying competitive levels and training backgrounds [19,35]. Other research has similarly emphasized that high levels of rapid force production are essential for reaching elite status [21,35]. Superior vertical jump performance provides athletes with an advantage during offensive actions, where the ability to elevate rapidly and to the greatest possible height enhances shooting success [2]. The difference observed between national team and non-national team players in our study suggests that jump performance may play a key role in talent identification and in establishing performance profiles in youth handball.

The 20 m sprint test also revealed significant differences between groups. National team players were faster, suggesting that linear acceleration capacity is a relevant performance determinant in youth female handball. Numerous studies support our findings, reporting superior sprint performance in top-level, compared to lower-level, athletes [34,36]. However, the literature is not entirely consistent, as several studies have shown that the linear sprint time does not always reliably differentiate between performance levels [19,37,38]. Overall, although our data and several previous studies identify 20 m sprint performance as a potential limiting factor, it may be beneficial to report additional details, such as age group, sample size, sprint distance, and differences in test protocols. Therefore, assessing linear speed alongside other measures of explosive strength is recommended.

Furthermore, our results showed no differences between groups in maximal strength, endurance performance, or change in direction ability. Concerning endurance performance, several reviews and empirical studies emphasize that intermittent aerobic tests and traditional VO2max assessments do not necessarily distinguish between elite and non-elite players in team sports. This may be linked to the sport-specific nature of aerobic demands, variability in testing protocols, and the limited association between laboratory VO2max values and match-specific physical outputs [39,40]. Change in direction performance is highly dependent on protocol characteristics (number and length of direction changes, angles, etc.), making comparisons across studies challenging. As highlighted by prior research, methodological differences alone may lead to inconsistent findings regarding performance differences across competitive levels [41]. The absence of significant differences in maximal strength measures (IMTP and relative bench press) was unexpected. However, several factors may explain this. The previous literature suggests that the relationship between one-repetition maximum measures and handball-specific performance is often mediated by fat-free mass [42]. Given the similarity between our groups in anthropometric and body composition profiles, differences in absolute force production may have been attenuated. Additionally, explosive strength expressed in CMJ performance does not necessarily correspond to maximal isometric strength, as the IMTP measures peak isometric force, whereas the CMJ reflects SSC efficiency and dynamic explosive power [43,44].

Regarding the fundamental anthropometric and body composition variables (body height and body mass), no significant differences were observed between the two groups. This suggests that, within this age category, these variables alone may not serve as distinguishing factors. In contrast, several classical and contemporary studies have reported opposite findings, particularly in younger or still developing age groups [20,44,45,46]. Earlier research demonstrated that, during talent identification in youth handball, greater body mass, taller stature, and more favorable body composition often accompany a higher competitive level [20,47]. Similarly, a study examining male handball players confirmed that, over the long term, a clear anthropometric trend emerges among world-class athletes, with pivots and defensive players being generally taller and possessing a larger body frame [45]. Overall, our findings suggest that although a substantial body of literature indicates that anthropometric characteristics may distinguish elite from non-elite athletes, in this sample of youth athletes these variables were not decisive. This may imply that, at this developmental stage, selection processes are primarily performance based rather than driven by anthropometric criteria.

Our findings hold substantial practical relevance for youth athlete development and performance diagnostics. The study identifies which conditional variables most strongly contribute to reaching the national team level and which objective markers are less suitable for distinguishing between competitive levels. These insights can inform and optimize talent identification and long-term athlete development models. Motor tests that showed significant differences in our study (e.g., explosive strength and speed) may serve as key indicators of advancement programs. Conversely, parameters that showed no group differences highlight the importance of avoiding a narrow anthropometric selection approach and instead adopting a broader, multifactorial performance profiling strategy. Our results thus provide practical guidance for coaches and sport scientists seeking to support the athletic development of youth handball players more effectively.

This study has several limitations that should be considered when interpreting the results. First, the relatively small sample size may limit the ability to detect subtle but practically relevant differences. Second, all tests were performed on a single occasion, which does not account for day-to-day fluctuations in readiness or fatigue. Additionally, training load and athletes training histories, were not controlled, which may have influenced performance outcomes. A limitation of the study is the lack of information on the athletes’ training volume and training type prior to selection, which prevents a clear distinction between whether the observed performance differences are causes or consequences of selection; nevertheless, the findings suggest that targeted development of these parameters may potentially improve selection chances. As such, caution is warranted when generalizing the finding, and further research with larger samples and longitudinal designs is required to confirm our conclusions.

Future studies should incorporate additional tests assessing psychological and cognitive functions, as these may play important roles in match performance. Furthermore, expanding the research to male youth athletes and exploring performance differences across distinct age categories may provide additional valuable insights.

## 5. Conclusions

The findings of this study indicate that selection to the youth national team level in female handball is primarily associated with superior explosive power and linear sprint performance rather than anthropometric characteristics or general strength and endurance measures. From a practical perspective, this suggests that regular monitoring and targeted development of jump performance and acceleration should be prioritized in youth training and talent identification processes, as these qualities are closely related to decisive match actions. Body composition variables alone appear to have limited discriminatory value at this age, emphasizing that selection decisions should be based mainly on objective, performance-related indicators. Overall, the results provide applied guidance for coaches and practitioners when defining development priorities in youth handball programs.

## Figures and Tables

**Table 1:** The mean and standard deviation values of the test results and basic anthropometric characteristic of the examined groups. N.T.A. = selected athletes, N.N.T.A. = non-selected athletes.

	N.T.A.		N.N.T.A.	
	Mean	SD	Mean	SD
Body height (cm)	171.35	4.73	172.01	8.30
Body mass (kg)	67.68	6.22	66.52	7.72
Skeletal muscle mass (kg)	31.19	3.31	30.31	4.06
Body fat percentage (%)	17.33	3.99	19.92	4.97
Jump height (cm)	41.17	3.56	36.32	3.01
Relative peak power (W/kg)	51.54	4.07	44.41	4.61
20 m sprint (s)	3.35	0.10	3.46	0.09
5-10-5 CODS (s)	4.93	0.14	4.98	0.11
IMTP (N)	2462.94	293.74	2476.54	476.03
Relative IMTP (N·kg^−1^)	36.53	4.58	35.11	10.50
Bench press (kg/ttkg)	0.78	0.12	0.71	0.11
YoYo IRL1 (m)	2100.00	337.26	1897.65	425.79

## Data Availability

The original contributions presented in this study are included in the article. Further inquiries can be directed at the corresponding author.

## Abbreviations

The following abbreviations are used in this manuscript:

CMJ	Countermovement Jump
IMTP	Isometric Mid-Thigh Pull
N.T.A.	National Team Athletes
N.N.T.A.	Non-national Team Athletes
CODS	Change in direction speed
IRL1	Intermittent Recovery Level 1
1RM	One-repetition Maximum
SSC	Stretch Shortening Cycle
